# Supplementary material for: BCG activation of trained immunity is associated with induction of cross reactive COVID-19 antibodies in a BCG vaccinated population
Source: PLoS One. 2024 May 9;19(5):e0302722. doi: 10.1371/journal.pone.0302722 (PMC11081370; doi:10.1371/journal.pone.0302722)
Supplement: S1 Fig — (DOCX) [file pone.0302722.s001.docx]

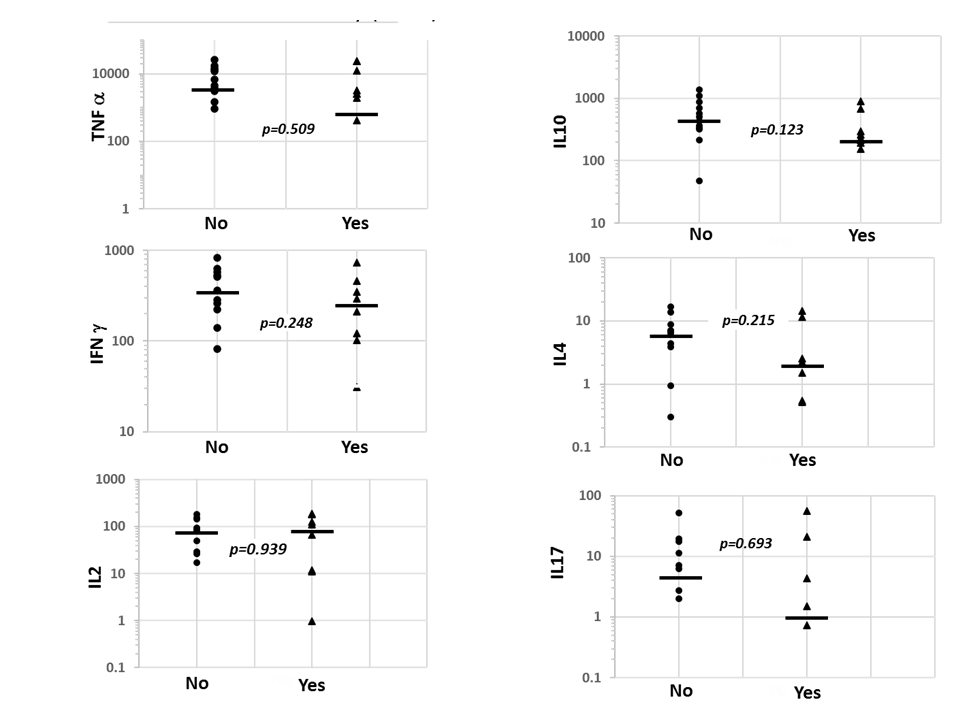


**S1 Fig. Comparison of cytokine secretion in relation to Bacille Calmette-Guerin (BCG) status in whole blood assay (WBA)**

Comparison of cytokine secretion in BCG scar positive (n=8) and scar negative (n=12) Scatter plot shows individual responses of cytokines in both groups. Results are shown as individual data points for stimulating cytokines in whole blood assay (WBA). The Mann-Whitney U test was applied to compare Scar positive and negative groups. The solid bars compare the median responses in the two groups.
