# Supplementary material for: BCG activation of trained immunity is associated with induction of cross reactive COVID-19 antibodies in a BCG vaccinated population
Source: PLoS One. 2024 May 9;19(5):e0302722. doi: 10.1371/journal.pone.0302722 (PMC11081370; doi:10.1371/journal.pone.0302722)
Supplement: S2 Fig — (DOCX) [file pone.0302722.s002.docx]

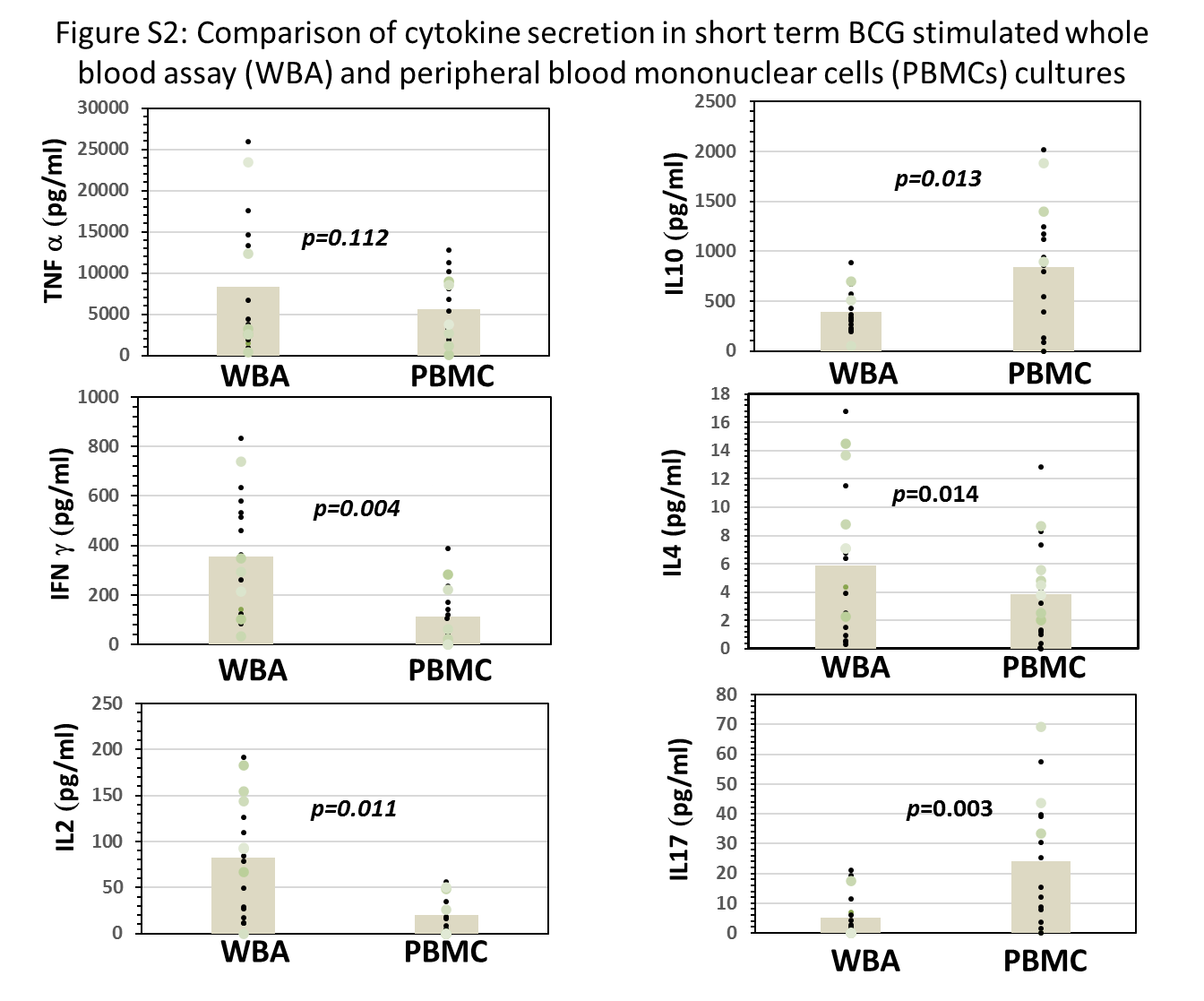


**S2 Fig. Comparison of cytokine secretion in short term BCG stimulated whole blood assay (WBA) and peripheral blood mononuclear cells (PBMCs) cultures.**

Comparison of cytokine secretion in short-term WBA (12 hrs.) and PBMC (12 hrs.) BCG stimulated cultures (n=20)**.** Whole blood was diluted 1/2 in complete RPMI media and stimulated with 60ul of BCG (2-8 x10^6 CFU/vial), to obtain an MOI of 1.2. For PBMC a concentration of ~2 million cells were used for the stimulation of cells. The results are shown as log values after deducting spontaneous secretion (pg/ml). The shaded bars indicate the mean levels. The Wilcoxon Sign Rank test was applied for the significant difference in the cytokine level. p<0.05 was considered significant.
