# Supplementary material for: BCG activation of trained immunity is associated with induction of cross reactive COVID-19 antibodies in a BCG vaccinated population
Source: PLoS One. 2024 May 9;19(5):e0302722. doi: 10.1371/journal.pone.0302722 (PMC11081370; doi:10.1371/journal.pone.0302722)
Supplement: S3 Fig — (DOCX) [file pone.0302722.s003.docx]

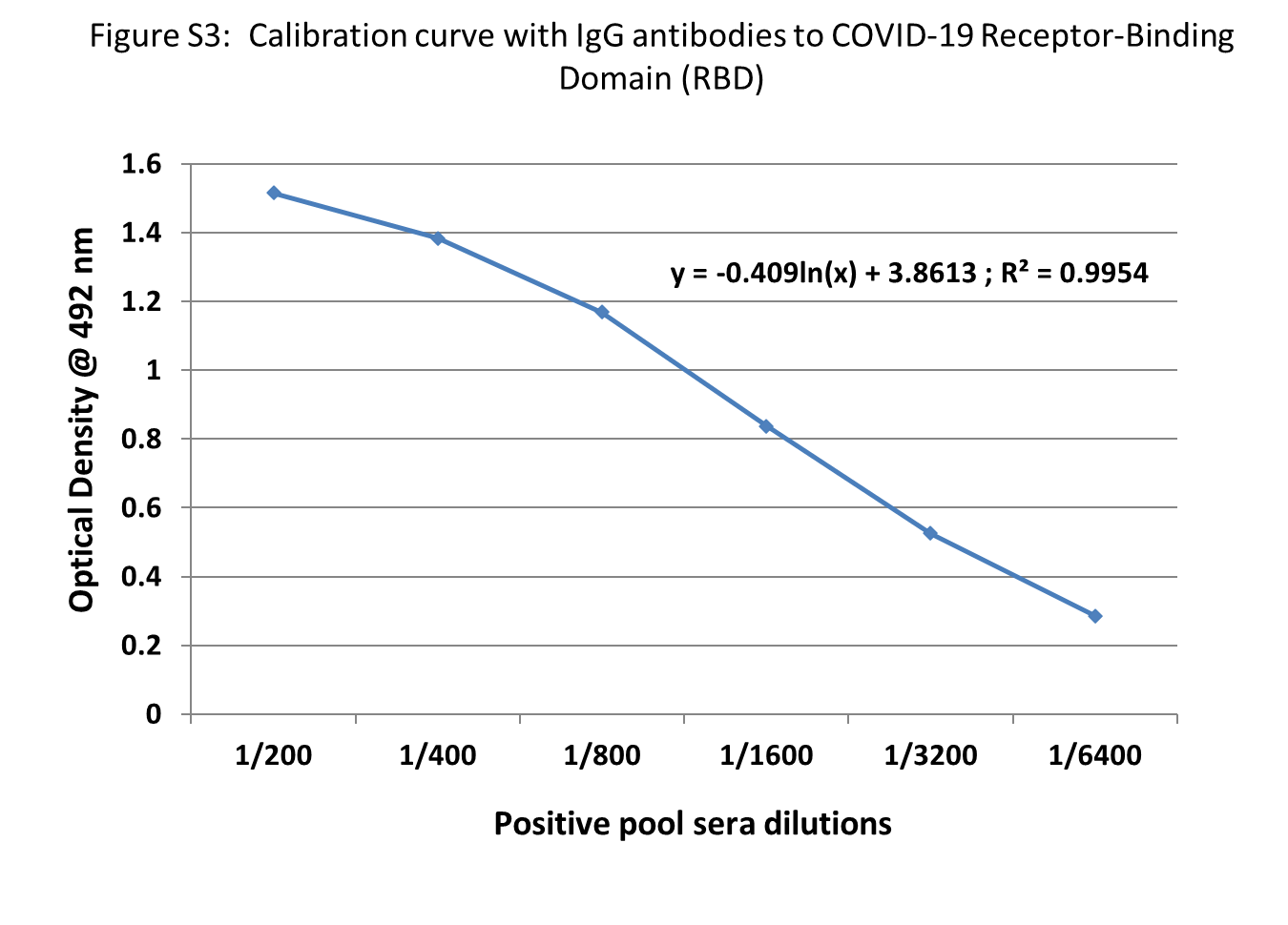


**Supplementary S3 Fig. Calibration curve with IgG antibodies to COVID-19 Receptor binding domain (RBD)**

A positive pool for IgG anti-RBD antibodies was diluted to determine endpoint titer. Endpoint titer was taken as one unit of antibody activity.
