# Supplementary material for: BCG activation of trained immunity is associated with induction of cross reactive COVID-19 antibodies in a BCG vaccinated population
Source: PLoS One. 2024 May 9;19(5):e0302722. doi: 10.1371/journal.pone.0302722 (PMC11081370; doi:10.1371/journal.pone.0302722)
Supplement: S1 Table — (DOCX) [file pone.0302722.s004.docx]

**S1 Table: Cells and inducers of cytokines in the Innate arm of the immune system**

| Subset of Immune cells | Inducers | Cytokines secreted |
| --- | --- | --- |
| M1 (inos) ^a^ | LPS, TNF | TNFα, IFNγ |
| M2A (arg) ^a^ | IL4 | IL10 |
| M2B (inos) ^a^ | LPS | IL10, TNFα |
| M2C (arg) ^a^ | IL10 | IL10 |
| γδ T cells (BTN3A2) ^b^ | BCG stress proteins LPS | IL4, IL17 |
| NK cells (GZMA) ^c^ | LPS, BCG | IL2, IFNγ, TNFα, IL10 |

a [1, 2], b [3, 4], c [5]

**Note: Cytokine and inducers analyzed in the current study**

**References:**

1. Foey AD. Macrophages—masters of immune activation, suppression and deviation. Immune response activation. 2014;276.

2. Martinez FO, Gordon S. The M1 and M2 paradigm of macrophage activation: time for reassessment. F1000prime reports. 2014;6.

3. Hoft DF, Brown RM, Roodman ST. Bacille Calmette-Guérin vaccination enhances human γδ T cell responsiveness to mycobacteria suggestive of a memory-like phenotype. The Journal of Immunology. 1998;161(2):1045-54.

4. Wo J, Zhang F, Li Z, Sun C, Zhang W, Sun G. The role of gamma-delta T cells in diseases of the central nervous system. Frontiers in immunology. 2020;11:580304.

5. Kleinnijenhuis J, Quintin J, Preijers F, Joosten LA, Jacobs C, Xavier RJ, et al. BCG-induced trained immunity in NK cells: role for non-specific protection to infection. Clinical immunology. 2014;155(2):213-9.
