# Supplementary material for: BCG activation of trained immunity is associated with induction of cross reactive COVID-19 antibodies in a BCG vaccinated population
Source: PLoS One. 2024 May 9;19(5):e0302722. doi: 10.1371/journal.pone.0302722 (PMC11081370; doi:10.1371/journal.pone.0302722)
Supplement: S2 Table — (DOCX) [file pone.0302722.s005.docx]

**S2 Table: Age-wise (minimum to maximum) BCG scar status, Mantoux Test (MT) readings, and gender distribution of study participants**

| S # | Participant ID | Age (years) | Gender | BCG Scar | MT reading (mm) |
| --- | --- | --- | --- | --- | --- |
| 1 | SMG-20 | 16 | Female | Yes | 0 |
| 2 | SMG-21 | 16 | Female | No | 7 |
| 3 | SMG-12 | 17 | Male | Yes | 0 |
| 4 | SMG-13 | 17 | Male | No | 2 |
| 5 | SMG-22 | 20 | Male | No | 0 |
| 6 | SMG-23 | 20 | Male | Yes | 0 |
| 7 | SMG-10 | 23 | Female | Yes | 0 |
| 8 | SMG-11 | 23 | Female | No | 0 |
| 9 | SMG-24 | 27 | Male | Yes | 0 |
| 10 | SMG-25 | 27 | Male | No | 0 |
| 11 | SMG-14 | 28 | Female | No | 0 |
| 12 | SMG-15 | 28 | Female | Yes | 4 |
| 13 | SMG-18 | 28 | Female | Yes | 0 |
| 14 | SMG-19 | 28 | Female | No | 0 |
| 15 | SMG-26 | 34 | Male | No | 0 |
| 16 | SMG-27 | 34 | Male | No | 0 |
| 17 | SMG-16 | 35 | Female | Yes | 0 |
| 18 | SMG-17 | 35 | Female | No | 0 |
| 19 | SMG-28 | 40 | Male | No | 0 |
| 20 | SMG-29 | 40 | Male | No | 0 |
