# Supplementary material for: BCG activation of trained immunity is associated with induction of cross reactive COVID-19 antibodies in a BCG vaccinated population
Source: PLoS One. 2024 May 9;19(5):e0302722. doi: 10.1371/journal.pone.0302722 (PMC11081370; doi:10.1371/journal.pone.0302722)
Supplement: S3 Table — (DOCX) [file pone.0302722.s006.docx]

**S3 Table: Primers for detecting gene expression in BCG-activated cells of the innate immune system.**

| S No | Target Gene* | Primer Sequence (5’ 3’) |
| --- | --- | --- |
| 1 | GZMA | Forward: TTT CTG GCA TCC TCT CTC TCA |
|  |  | Reverse: GGG TCA TAG CAT GGA TAG GG |
| 2 | BTN3A2 | Forward: AAG ACA GCC AGC ATT TCC AT |
|  |  | Reverse: GAG AAG CAG CAG CAA GAT AGG |
| 3 | TNFα | Forward: AGC CCA TGT TGT AGC AAA CC |
|  |  | Reverse: TGA GGT ACA GGC CCT CTG AT |
| 4 | 36B4 | Forward: TCC TCT CAC CAG GTG TCG TC |
|  |  | Reverse: CTG TCT TCC CTG GGC ATC AC |

*****GZMA (Granzyme A); marker for natural killer (NK) cells. BTN (Butyrophilin); marker for gamma-delta (γδ) T cells. TNFα (Tumor Necrosis Factor); marker for M1 cells
