# Supplementary material for: BCG activation of trained immunity is associated with induction of cross reactive COVID-19 antibodies in a BCG vaccinated population
Source: PLoS One. 2024 May 9;19(5):e0302722. doi: 10.1371/journal.pone.0302722 (PMC11081370; doi:10.1371/journal.pone.0302722)
Supplement: S4 Table — (DOCX) [file pone.0302722.s007.docx]

**S4 Table. Comparison of spontaneous secretion of cytokines in whole blood assay (WBA) and peripheral blood mononuclear cells (PBMCs) cultures**

| Cytokines (pg/ml) * | WBA (n=20) | PBMC (n=20) | *p-*value ^ɸ^ |
| --- | --- | --- | --- |
| IL-2 (mean ± SE) | 3.2 ± 2.8 | 0.46 ± 0.20 | 0.500 |
| IL-4 (mean ± SE) | 0.09 ± 0.06 | 0.04 ± 0.02 | 0.859 |
| IL-17(mean ± SE) | 0.03 ± 0.03 | 0.10 ± 0.10 | 0.317 |
| IL-10 (mean ± SE) | 12.1 ± 9.1 | 15.3 ± 10.6 | 0.017 |
| IFNγ (mean ± SE) | 12.7 ± 7.7 | 8.7 ± 5.4 | 0.715 |
| TNFα (mean ± SE) | 69.2 ± 67.1 | 50.0 ± 23.9 | 0.009 |

***** IL, interleukin; SE, standard error; IFNγ, interferon-gamma; TNFα, tumor necrosis factor-alpha

^ɸ^ *p* value <0.05 considered a significant value

Cytokine mean levels and standard error around the means are given. Cytokines were assessed by the Luminex assay system. Wilcoxon sign rank test was applied for the significant difference in spontaneous secretion of cytokines in WBA (12hrs) and PBMC (12hrs) cultures. The Mann-Whitney U test was carried out to determine the significance of differences in the two assays.
