# Supplementary material for: BCG activation of trained immunity is associated with induction of cross reactive COVID-19 antibodies in a BCG vaccinated population
Source: PLoS One. 2024 May 9;19(5):e0302722. doi: 10.1371/journal.pone.0302722 (PMC11081370; doi:10.1371/journal.pone.0302722)
Supplement: S5 Table — (DOCX) [file pone.0302722.s008.docx]

**S5 Table. The magnitude of BCG recall responses in Trained Immunity (TI)**

| Cytokines* | WBA ratio (stimulated / spontaneous) | PBMCs ratio (stimulated / spontaneous) |
| --- | --- | --- |
| TNFα | 132 (9148 / 69) | 113 (5640 / 50) |
| IFNγ | 27 (355 / 13) | 132 (132 / 1) |
| IL-10 | 40 (488 / 12) | 56 (842 / 15) |
| IL-2 | 27 (83 / 3) | 24 (24/ 1) |
| IL-4 | 6 (6 / 1) | 4 (4 / 1) |
| IL-17 | 10 (10 / 1) | 24 (24 / 1) |

* TNFα, tumor necrosis factor alpha; IFNγ, interferon gamma; IL, interleukin

BCG recall responses in whole blood assay (WBA) and peripheral blood mononuclear cells (PBMCs) were determined by the ratio of stimulated cytokines levels divided by spontaneous cytokines levels. M1 derived cytokines (TNFα, IFNγ); M2 derived (IL10); NK cells (IL2), gamma-delta (γδ) T cells (IL4, IL17), (refer to supplementary Table S1). Cytokines <1.0 pg/ml were rounded off to 1.0.
