# Supplementary material for: BCG activation of trained immunity is associated with induction of cross reactive COVID-19 antibodies in a BCG vaccinated population
Source: PLoS One. 2024 May 9;19(5):e0302722. doi: 10.1371/journal.pone.0302722 (PMC11081370; doi:10.1371/journal.pone.0302722)
Supplement: S6 Table — (DOCX) [file pone.0302722.s009.docx]

**S6 Table. Correlation between cytokines secretions in response to Bacille Calmette-Guerin in Whole Blood Assay (WBA) culture**

| Cytokines Correlation Coefficient | IL2 BCG_12hrs WBA | IL4 BCG_12hrs WBA | IL10 BCG_12hrs WBA | IL17 BCG_12hrs WBA | IFNγ BCG_12hrs WBA | TNFα BCG_12hrs WBA |
| --- | --- | --- | --- | --- | --- | --- |
| IL2 BCG_12hrs WBA | 1.000 | .642^**^ | .277 | .676^**^ | .674^**^ | .642^**^ |
| IL4 BCG_12hrs WBA | .642^**^ | 1.000 | .515^*^ | .647^**^ | .838^**^ | .860^**^ |
| IL10 BCG_12hrs WBA | .277 | .515^*^ | 1.000 | .361 | .367 | .362 |
| IL17 BCG_12hrs WBA | .676^**^ | .647^**^ | .361 | 1.000 | .711^**^ | .528^*^ |
| IFNγ BCG_12hrs WBA | .674^**^ | .838^**^ | .367 | .711^**^ | 1.000 | .759^**^ |
| TNFα BCG_12hrs WBA | .642^**^ | .860^**^ | .362 | .528^*^ | .759^**^ | 1.000 |

** Correlation is significant at the 0.01 level (2-tailed)

* Correlation is significant at the 0.05 level (2-tailed)
