# Supplementary material for: BCG activation of trained immunity is associated with induction of cross reactive COVID-19 antibodies in a BCG vaccinated population
Source: PLoS One. 2024 May 9;19(5):e0302722. doi: 10.1371/journal.pone.0302722 (PMC11081370; doi:10.1371/journal.pone.0302722)
Supplement: S7 Table — (DOCX) [file pone.0302722.s010.docx]

**S7 Table. Correlation between cytokine secretions in response to Bacille Calmette-Guerin in Peripheral Blood Mononuclear Cells (PBMCs) culture**

| Cytokines Correlation Coefficient | IL2 BCG_48hrs PBMC | IL4 BCG_48hrs PBMC | IL10 BCG_48hrs PBMC | IL17 BCG_48hrs PBMC | IFNγ BCG_48hrs PBMC | TNFα BCG_48hrs PBMC |
| --- | --- | --- | --- | --- | --- | --- |
| IL10 BCG_48hrs PBMC | .599* | .570* | 1.000 | .681** | .455 | .541* |
| IL17 BCG_48hrs PBMC | .744** | .941** | .681** | 1.000 | .862** | .911** |
| IFNγ BCG_48hrs PBMC | .717** | .923** | .455 | .862** | 1.000 | .961** |
| TNFα BCG_48hrs PBMC | .696** | .950** | .541* | .911** | .961** | 1.000 |
| IL4 BCG_48hrs PBMC | .700** | 1.000 | .570* | .941** | .923** | .950** |

** Correlation is significant at the 0.01 level (2-tailed)

* Correlation is significant at the 0.05 level (2-tailed)
