# Supplementary material for: BCG activation of trained immunity is associated with induction of cross reactive COVID-19 antibodies in a BCG vaccinated population
Source: PLoS One. 2024 May 9;19(5):e0302722. doi: 10.1371/journal.pone.0302722 (PMC11081370; doi:10.1371/journal.pone.0302722)
Supplement: S8 Table — (DOCX) [file pone.0302722.s011.docx]

**S8 Table. Spearman Rank correlation between BCG and LPS stimulated cytokines secreted in whole blood assay (WBA) culture.**

| BCG vs LPS | IL2 | IL4 | IL10 | IL17 | IFNγ | TNFα |
| --- | --- | --- | --- | --- | --- | --- |
| rho | 0.562* | 0.611** | -0.033 | 0.273 | 0.464* | 0.670** |
| *p* | 0.015 | 0.005 | 0.896 | 0.258 | 0.045 | 0.002 |
| n | 18 | 19 | 18 | 19 | 19 | 19 |

** Correlation is significant at the 0.01 level (2-tailed)

* Correlation is significant at the 0.05 level (2-tailed)

*p-*value <0.05 considered as significant value.
